# Tobacco Taxation Influences the Smoking Habits of Adult Smokers Attending Smoking Cessation Clinic in Saudi Arabia

**DOI:** 10.3389/fpubh.2022.794237

**Published:** 2022-02-21

**Authors:** Najeeb Saud S. Altowiher, Rami Bustami, Ali M. Alwadey, Mansour Alqahtani

**Affiliations:** ^1^Tobacco Control Program, Ministry of Health (Saudi Arabia), Riyadh, Saudi Arabia; ^2^Department Biostatistics and Epidemiology, College of Medicine, Alfaisal University, Riyadh, Saudi Arabia

**Keywords:** tobacco taxation, tobacco control, smoking cessation, public health, health policy

## Abstract

**Objectives:**

To determine whether the increased tobacco price due to tax implementation on tobacco products (including cigarettes) has a significant effect on smoking cessation among Saudi Arabian adult smokers.

**Methods:**

An interviewer-administered questionnaire was used to obtain data from adult Saudi smokers and recent quitters attending smoking cessation clinics between January 2018 and September 2019. The responses of the participants were summarized and analyzed.

**Results:**

In total, 660 participants were interviewed, of which 98% were men who resided in the western region (33%). Taxation had no effect on smoking in 387 participants [58.6%; 95% confidence interval (CI): 54.9, 62.4], some effect in 220 participants (33.3%; 95% CI: 29.7, 36.9), and a substantial effect in 50 participants (7.6%; 95% CI: 5.6, 9.6). Strategies adopted to cope with the tax implementation included cutting down on the number of cigarettes smoked (302; 45.8%), changing to a cheaper brand of cigarette (151; 22.9%), purchasing in bulk (105; 15.9%), attempting to quit (453; 68.6%), and doing nothing (108; 16.4%). The rate of quitting smoking after attending the clinic was 20.7% (95% CI: 17.7, 23.9). Occupation (*P* = 0.003), education (*P* = 0.03), and current smoking habit (*P* = 0.07) were significantly associated with the impact of tobacco taxation. The strategies adopted in response to tax implementation on cigarettes were significantly associated with occupation (χ^2^ = 30, degrees of freedom = 12, *P* < 0.001).

**Conclusions:**

Tobacco taxation influenced 40% of the participants. Their attempts to opt for alternatives should be recognized in evaluating policies to reduce adverse health impacts caused by tobacco abuse.

## Introduction

### Background to the Study

Tobacco use is the leading cause of 6 million annual deaths worldwide; however, the global tobacco epidemic has reached significant levels, with ~1.3 billion tobacco users (1). The daily prevalence of smoking in 2017 was 25% (2). Given its negative effects on health, society, and the economy, the World Health Assembly unanimously adopted the World Health Organization Framework Convention on Tobacco Control (WHO FCTC) to reduce the tobacco epidemic's globalization and its severe consequences on public health. The WHO FCTC assists nations by introducing six evidence-based tobacco reduction measures known as the MPOWER package. As per the Convention, the term MPOWER refers to M: monitoring tobacco use and prevention policies; P: protecting people from tobacco smoke; O: offering help in quitting tobacco use; W: warning individuals about the dangers of tobacco; E: enforcing bans on tobacco advertising, promotion, and sponsorship; and R: Raising taxes on tobacco (3). Smoking has been progressively prevalent in Saudi Arabia for decades. Nationwide cross-sectional surveys conducted in 2018 across the 13 regions of Saudi Arabia revealed a 21.4% prevalence of cigarette smoking. The same study also revealed that cigarette smoking was more common among males (32.5%) than females (3.9%) (4). An earlier national survey conducted in 2013 showed that the overall prevalence of current smoking was 15.3%, and smoking prevalence was still higher among males than females (28 vs. 1.9%) (5). A similar study conducted in 2005 revealed that the prevalence of cigarette smoking was 12.2%, still being more prevalent among males (23.6%) than females (1.5%) (6) based on these statistics. It is evident that cigarette smoking has been increasing gradually since the turn of the decade. It can also be noted that there has been a near-constant difference between males and females regarding cigarette smoking with far more males being prone to smoking compared to females.

### Relative Effectiveness of Tobacco Taxation

According to Article 6 of the WHO FCTC, price and tax measures effectively reduce tobacco use. Countries are responsible for setting, implementing, and maintaining tax policies (1). Positive impacts of increasing the price of tobacco products through taxation have been found in developing and industrialized countries/regions, such as Australia, the United States of America (US), and European countries. Therefore, it is expected that similar results can be or have already been achieved in Saudi Arabia. For instance, in 2014, a study assessing behavioral change in smokers after a tax increase from 70 to 95% in Minnesota, US revealed that ~38% of the participants reported behavioral changes related to the tax (an attempt at quitting or reducing). Moreover, the study demonstrated that the most significant changes were more common among daily smokers (46%) than others (7).

Similarly, a study was conducted assessing the impact of a simulated 10% tax-induced cigarette price increase across 36 European countries. It demonstrated an overall 3.1% drop in the total cigarette consumption (8). Correspondingly, in New South Wales, Australia, a study on recent tobacco tax increase found that the most frequent response among participants was “cutting down on the number of cigarettes smoked.” Moreover, the tax increase had an impact on quitting. Quitting after the tax increase was significantly more common than quitting before the tax increase (9). In early 2015, the Republic of Korea increased taxes on cigarettes by 80%. A study conducted that same year showed that 36.1% of the participants reported a change in their behavior after increased taxation. Furthermore, among current smokers, 25% stated that they had reduced the number of cigarettes they smoked. Among recent quitters, 39% stated that they quit due to the tax increase (10). Tobacco taxation is considered the most effective intervention policy to reduce the demand for tobacco across all socioeconomics groups. Lower-income smokers are most affected by such policies (9, 11). For example, higher rates of action are observed in response to the tobacco tax increase among lower socioeconomic groups than among individuals within medium/high socioeconomic groups living in Minnesota, US (12). Studies have shown that smoking is typically high among low-income groups in the majority of developed countries. For instance, in the US, over 30% of people living below poverty are smokers. Moreover, in the United Kingdom, smoking rates are higher in disadvantaged neighborhoods with high unemployment levels (13). Therefore, most researchers have suggested that the revenue derived from the increased price should be used to help low-income smokers quit, avoiding equity implications (11).

The Eastern Mediterranean Region of the WHO consists of 22 countries, which vary in their levels of income. Tobacco consumption is expected to grow by 25% in 2025, despite a reduction in Asia, North America, and Europe (14). According to a study conducted between 2015 and 2017, which evaluated the progress and challenges in implementing the MPOWER policy measures in the region, tobacco taxation was considered unsuccessful, even in countries with high score for other measures. One recommendation to achieve an overall improvement in effective tobacco control measures in this region was to implement taxation (14). Nevertheless, since the publication of this study, some countries in the region have implemented tobacco taxation.

Based on the foregoing statistics, cigarette smoking and tobacco use are rather rampant in Saudi Arabia. Since 2005, when smoking and/or tobacco consumption statistics were made available through empirical research, there has been a steady increase in the number of people smoking cigarettes or using tobacco. Consequently, the Saudi Ministry of Health established a tobacco control program in 2002. The Tobacco Control Program is a government-sponsored program that develops regulations, policies, and procedures for tobacco control. It provides multiple services, including raising awareness through multiple channels, establishing clinics for smoking cessation around the Kingdom as a part of the national health care system wherein the government provides free health care services and most importantly, gathering data on the smoking status in Saudi Arabia (15). Despite these efforts designed to help reduce tobacco consumption, the most recent nationwide cross-sectional surveys conducted in 2018 showed an increased prevalence of smoking. Thus, Saudi Arabia ratified the Framework Convention on Tobacco Control and enforced a tobacco taxation policy in the second quarter of 2017, with a total tax of 100% (16, 17). However, only a few studies have been conducted to evaluate how taxation affects smoking habits demonstrating the effect of policies promoted by such a program (15, 18–20); this is due to the lack of adequate empirical research on the effect of tobacco taxes on smoking cessation in the Saudi Arabian context. Nevertheless, such studies have been conducted in other parts of the world (especially in the developed Western World). Thus, the results may vary compared to those of other countries in the West.

## Methods

### Ethics, Study Participants, and Study Procedure

This study was approved by the Saudi ministry of health Institutional Research and Ethical Committee. Data were gathered by the Tobacco Control Program of the Ministry of Health, Saudi Arabia. Details of the study explained to all participants, personal information was not required or used in the study. The consent form was read and verbally explained to all participants in order to ensure that each one of them took part in the study based on their informed consent and not out of coercion or compulsion. Participants were ensured that participation in the study (and especially in the questionnaire surveys) was voluntary and involvement in the survey implied the participants' consent to the study. All participants were that their participation and roles in the study would be anonymous and confidential.

All tenets of the Declaration of Helsinki were strictly adhered to throughout the research. Saudi Arabian government has implemented a 100% tobacco taxation (including cigarettes) policy in the second quarter of 2017 (16, 17). Therefore, a population of any Saudi adult smokers attending smoking cessation clinics between January 2018 and September 2019 were included in the study. Meanwhile, children, none smokers and residents from other nationalities were excluded from the study.

A total of 110,925 adult smokers attended 20 smoking cessation clinics under the tobacco control program during the study period (15). Based on previous studies we assumed that 27% of 110,925 smokers tried changing their smoking approach to quit smoking because of the taxation of tobacco products (9). To achieve a 95% confidence interval (CI), 5% acceptable margin of error, and clustering effect of 2, we needed a sample size of 605. We used the Stat calculator of the Open Epi software to calculate the sample size for a cross-sectional survey (21). To compensate for non-response, we increased the sample size by 10%. Finally, 660 participants were included. The national sample of clinic attendees was stratified by clinic attendance in the five zones of Saudi Arabia.

The attendees of 20 clinics provided from the Tobacco control program were listed into a Microsoft Excel spreadsheet, and stratified random sampling method was used to select the required sample in each zone proportionally.

A physician conducted telephonic interviews in Arabic with adult smokers and recent quitters for participants recruited to the telephone survey using simple random sampling through the Excel spreadsheet provided by from the Tobacco control program.

A pilot study was performed to test the survey tool which was altered before the main survey to improve its effectiveness.

Demographic information of the participants included age, sex, education, occupation, and income as well as their clinic location. Participants were grouped as follows based on their reported age: 18–29 years, 30–54 years, and 55 years or older. Monthly income was converted from riyals to US dollars considering 1,000 US $ = 3,750 SR. Regarding education, the participants were grouped as, “school graduates, college graduates, and graduates with degree higher than a college degree.” Occupation was grouped as unemployed, retired, and public and private sector jobs. The questionnaire included aspects on current smoking status, the purpose of attending the smoking cessation clinic.

Participants were asked current smoking status, the purpose of attending the smoking cessation clinic. Participants were asked what effect, if any, the increasing price of cigarettes had on them when: (a) cut down of consumption; (b) changed to lower price brand; (c) bought in bulk; (d) tried quitting; or (e) no change. Multiple responses were allowed. Participants were asked how much the increasing price of cigarettes affected them to quit (a) no effect; (b) some effect; or (c) great effect.

### Statistical Analysis

Data were collected on a pretested data collection form and transferred to the Statistical Package for Social Sciences spreadsheet (SPSS 25, IBM, NY, USA). Qualitative variables are presented as numbers and percentages. Quantitative variables are presented as the mean and standard deviation. To compare outcomes of the subgroups' variables, we estimated the chi-square values, degrees of freedom, and two-sided *P*-values. To study the interaction between the variables with significant associations of variables, we performed two-sided *P*-values. Statistical significance was set at *P* < 0.05.

## Results

We enrolled 660 participants in the survey. The number and proportion of participants in the five regions of Saudi Arabia and those attending smoking cessation clinics are shown in Table 1. The regional representation of participants was proportionately adequate.

**Table 1:** Proportion of population and survey participants in the five zones of Saudi Arabia.

**Region**	**Registered at smoking cessation clinic**		**Surveyed**	
	**Number**	**%**	**Number**	**%**
Central	23,438	22.1	139	21.2
Eastern	11,734	11.0	98	14.8
Northern	15,718	14.8	94	14.2
Western	36,345	34.2	215	32.6
Southern	19,018	17.9	113	17.1
Total	1,06,253	100	660	100

The profiles of the participants are listed in Table 2, of which majority were men (98%) and very few were uneducated (0.3%) or unemployed (12.1%). Thus, few individuals were in the higher income group (2%). In total, 387 participants (58.6%) responded that the increased price of tobacco due to taxation did not affect their smoking habits (95% CI: 54.9, 62.4).

**Table 2:** Profile of Saudi adults attending the smoking cessation clinic who participated in the survey.

**Age**	**Mean ± standard deviation**	**35.5 ± 9.4**	
	**Minimum, maximum**	**20, 71**	
		**Number**	**%**
Gender	Male	646	98
	Female	13	2
Education	Uneducated	3	0.3
	School graduate	313	47.4
	College graduate	324	49.1
	Higher education	20	3
Occupation	Unemployed	80	12.1
	Retired	52	7.9
	Govt employee	328	49.7
	Non-govt employee	200	30.3
Residents of Saudi Arabia	Central	139	21.1
	Eastern	98	14.8
	Northern	94	14.2
	Western	216	32.7
	Southern	113	17.1
Monthly income (US $)	<1,334	215	32.6
	1,334–2,667	287	43.5
	2,668–4,000	145	22
	≥ 4,001	13	2

Altogether, 220 adult smokers (33.3%) believed that the increased tobacco prices due to taxation partially affected their smoking habits (95% CI: 29.7, 36.9). However, only 50 smokers (7.6%) believed that taxation-based price increase had a significant effect on their smoking habits (95% CI: 5.6, 9.6).

The participants' responses to current smoking habits suggested that after attending the smoking cessation clinic, 137 (20.7%; 95% CI: 17.7, 23.9) quit smoking, while 523 (79.2%; 95% CI: 77.1, 83.2) continued smoking.

The association between the increased price of tobacco due to taxation and altered smoking habits by determinants is presented in Table 3. Occupation (*P* = 0.003), education (*P* = 0.03), and current smoking status (*P* = 0.07) were associated with the impact of increased tobacco prices. When these three factors were further analyzed, only current smoking status was a significant predictor of the negative impact of increased tobacco prices on smoking.

**Table 3:** Factors associated with the response to taxation in smoking cession motivation based on a univariate analysis.

		**No effect (*n* = 387)**		**Some effect (*n* = 220)**		**Great effect (*n* = 50)**		***P*-value**
		**Number**	**(%)**	**Number**	**(%)**	**Number**	**(%)**	
Sex	Male	377	97.4	218	99.1	50	100	*P* = 0.17
	Female	9	2.3	2	0.9	0	0	
Monthly income (US $)	Less than 1,334	125	32.3	68	30.9	16	32	*P* = 0.74
	1,334 to 2,667	161	41.6	103	46.8	23	46	
	2668 to 4,000	91	23.5	45	20.5	9	18	
	4,001 and more	8	2.1	3	1.4	2	4	
Occupation	Unemployed	52	13.4	20	9.1	5	10	χ^2^ = 19.2
	Retired	30	7.8	16	7.3	6	12	Df = 11
	Government employee	195	50.4	108	49.1	24	48	*P* = 0.003
	Non-government employee	109	28.2	75	34.1	15	30	
Education	Uneducated	1	0.3	0	0	1	2	χ^2^ = 13.2
	School graduate	193	49.9	91	41.4	29	58	Df = 11
	College graduate	178	46	124	56.4	20	40	*P* = 0.03
	Higher education	14	3.6	5	2.3	1	2	
Age		35.2		35.6		37.5		*P* = 0.27
		9.2		9.4		10.5	
Purpose to attend clinic	To quit	368	95.1	211	95.9	45	90	*P* = 0.5
	To reduce smoking	19	4.9	7	3.2	5	10	
Current smoking status	Quit	70	18.1	52	23.6	13	26	*P* = 0.07
	Smoking	317	81.9	168	76.4	37	74	
Zone of Saudi Arabia	Central	92	23.8	41	18.6	6	12	*P* = 0.03
	East	49	12.7	35	15.9	13	26	
	North	61	15.8	26	11.8	7	14	
	West	131	33.9	69	31.4	15	30	
	South	54	14	49	22.3	9	18	

*Statistically significant difference: P < 0.05*.

The change in smoking approach adopted to address the increased price of tobacco products due to taxation were as follows: 453 (68.6%) attempted to quit, 302 (45.8%) tried cutting down on the number of cigarettes smoked, 151 (23%) opted for a cheaper brand of tobacco products, 105 (16%) opted to buy in bulk, and 108 (16.4%) did not change their smoking habits. The majority of participants tried a combination of these strategies (Table 4). Occupation was significantly associated with variation in strategies (*P* < 0.001). The outcomes of our study were compared to those reported in the literature, especially at the regional level (Table 5).

**Table 4:** Change in smoking approach adopted in response to increased taxation on tobacco products by determinants.

		**Cut down**		**Change brand**		**Bought in bulk**		**Tried quitting**		**No change**		***P*-value**
		**Number**	**%**	**Number**	**%**	**Number**	**%**	**Number**	**%**	**Number**	**%**	
Sex	Male	299	46.5	151	23.5	105	16.3	446	69.4	104	16.2	χ^2^ = 4, df = 4, *P* = 0.04
	Female	3	25.0	0	0.0	0	0.0	7	58.3	4	33.3	
Age group	18–29	98	46.2	55	25.9	31	14.6	137	64.6	44	20.8	χ^2^ = 12, df = 8, *P* = 0.1
	30–54	193	45.5	91	21.5	74	17.5	298	70.3	66	15.6	
	55 <	11	52.4	6	28.6	0	0.0	20	95.2	1	4.8	
Education	School	140	44.4	77	24.4	45	14.3	213	67.6	57	18.1	χ^2^= 6, df = 8, *P* = 08
	College	154	47.5	69	21.3	56	17.3	228	70.4	49	15.1	
	Higher education	5	25.0	5	25.0	4	20.0	14	70.0	5	25.0	
Monthly income (US $)	> 1,334	87	41.0	58	27.4	30	14.2	135	63.7	48	22.6	χ^2^ = 17, df = 12, *P* = 0.1
	1,334–2,667	136	47.4	63	22.0	45	15.7	207	72.1	39	13.6	
	2,668–4,000	74	51.0	29	20.0	25	17.2	105	72.4	22	15.2	
	4,001 ≤	6	46.2	2	15.4	5	38.5	8	61.5	2	15.4	
Occupation	Unemployed	21	26.9	19	24.4	11	14.1	34	43.6	30	38.5	χ^2^ = 30, df = 12, *P* < 0.001
	Retired	26	50.0	12	23.1	5	9.6	39	75.0	6	11.5	
	Government employee	150	45.7	72	22.0	58	17.7	231	70.4	50	15.2	
	Non-government employee	105	52.5	49	24.5	30	15.0	151	75.5	26	13.0	

*Statistically significant difference: P < 0.05*.

**Table 5:** Impact of taxation on smoking cessation attempts of adult smokers in published studies and the present study.

**References**	**Country**	**Sample**	**Reduction of smoking**	**Change in approach**
Boyle et al. (7)	USA	1,382	15.6% quit	E-cigarette use
			60% attempted quitting	
Schafferer et al. (8)	36 European countries	–	3.1% quitting rate	Licit consumption rate decline by 18.4%
Dunlop et al. (9)	Australia	997	47.5% smoking changes	11.4% product-related changes
Han (10)	Korea	45,686	3.8% quit	22.8% reduced smoking
				5.4% switched to e cigarette
Park et al. (12)	USA	–	8% quit	40% altered smoking habit
AlGhamdi et al. (18)	Saudi Arabia	376	No change 39.6%	29.8% changed to cheaper brand
AlQarni (20)	Saudi Arabia	334	10% reduced consumption	20% switched to cheaper brand
				60% no change
Present study	Saudi Arabia	660	20% quit	68.6% attempted quitting
				45.8% reduced quantity
				23% switched to cheaper brand

## Discussion

In total, 40% of the participants believed that the increased price of tobacco had either substantial or some influence on their decision to visit the Saudi Ministry of Health's smoking cessation clinic, and more than half believed that the increased price of tobacco products had no influence on their decision to join the program or quit smoking. Similarly, one-third of the participants admitted that the increased price of tobacco products had a temporary effect, and only one in 12 participants believed it had a significant positive effect.

The participants of the present study were adult Saudi smokers willing to address their issues with tobacco consumption by attending smoking cessation clinics organized by the Ministry of Health. Our study participants understood their substance abuse issues and sought for help. Increasing taxation on tobacco products had a positive impact on only one-fifth of the participants. However, the willingness of all participants to reduce tobacco consumption could be a positive step in reaching the goal of quitting smoking in the future and can be explained by the increased prices of tobacco products or the attendance of smoking cessation clinics. Policymakers should therefore, focus on this population and support them by offering alternative smoking cessation strategies in addition to increasing the prices of tobacco products.

The smoking cessation rate in the present study compared with that of other studies shows that the increased prices due to taxation, which makes tobacco products expensive to the consumer, helped 20% of the adult smoking population to quit smoking, attempt to quit smoking, or reduce the smoking frequency (9, 10, 12). The option of switching to cheaper brands of tobacco products could be counterproductive and may cause more harm. For example, in Asian countries, the smoking of “biddies” (filter-less cigarettes) causes more concentrated fumes, which negatively affects the lung tissue and results in the development of fibrosis and communicable diseases such as tuberculosis (22, 23).

In our study, high education had a positive impact on both quitting/reducing smoking and adopting alternative strategies. The higher awareness of the negative effects of smoking among educated Saudi Arabians, as observed among the Polish, could explain their greater willingness and attempts to quit smoking compared to the uneducated people (24).

Government employees had significantly higher rates of maintaining their smoking habits and adopting alternative strategies. This could be due to better income and secured financial status; thus, the increased price of tobacco products had less effect on their smoking habits. This could also be explained by the positive impact of acculturation, which is more evident among those working in the private sector and outdoor sales jobs (25).

In our study, the female-to-male ratio was 1:50, which is well above the 1:38 ratio reported among Egyptian smokers (1). Considering young college-educated Saudi students, the smoking rate among women is 5% compared with 26% in men (26). Women may opt for alternative methods of tobacco consumption. A cessation rate of 20% in Saudi Arabia is of concern. Using nicotine patches (27), e-cigarettes (28), and intense health promotion are recommended strategies to reduce tobacco consumption for those attending smoking cessation clinics (29).

The findings of this study will effectively fill gaps in knowledge on the effectiveness of tobacco taxation in minimizing tobacco use. Targeting the entire population makes the findings easier to generalize. Therefore, this study has some limitations. First, the participants were recruited from smoking cessation clinics, which could have biased the findings because they were in favor of quitting/reducing smoking, and the findings of the present study may not be applicable to adult smokers who have not yet visited such clinics. Thus, a highly trained and skilled team is needed to survey a large population to obtain a representative result. However, to the best of our knowledge, adult Saudi smokers willing to quit and attend smoking cessation clinics have not yet been surveyed. Second, shisha is prevalent among young Saudi smokers (33% compared with 13% who smoke cigarettes) (30). Omitting the mode of tobacco consumption from the data obtained in this study limits the formulation and application of tobacco-related health policies. Third, we did not obtain the sex distribution of those attending the smoking cessation clinics; therefore, it is difficult to explain the low presentation of women in our study.

## Conclusion

The findings of our study showed the effect of the tobacco taxation policy during its first 3 years (approximately) of implementation among Saudi adult smokers attending smoking cessation clinics. However, a significant percentage of people did not reduce their smoking frequency or quit smoking. If the results could be generalized to the larger Saudi population, this would indicate that implementing a tobacco tax is one of the most critical effective measures in reducing smoking. Other strategies or policies should be considered to encourage smoking cessation. Considering the taxation policy, it is evident that it has been implemented as envisaged and has produced some intended outcomes in a short period based on the findings from the present study and previous literature (1, 7–9). However, policymakers may consider unifying all tobacco products and cigarette brands to the highest price after taxation. As well as, increasing the emphasis on surveillance and patience for the policy to reach its intended impact, and consumers change their perspective on increased taxation on tobacco products.

## Data Availability Statement

The original contributions presented in the study are included in the article/supplementary material, further inquiries can be directed to the corresponding author.

## Ethics Statement

The studies involving human participants were reviewed and approved by Ministry of Health IRB. The patients/participants were ensured that participation in the study (and especially in the questionnaire surveys) was voluntary and involvement in the survey implied the participants' consent to the study. All participants were that their participation and roles in the study would be anonymous and confidential.

## Author Contributions

NA: planning, data collection, field part, data management, and manuscript writing. RB: study design and revising manuscript. AA: data acquisition and result discussion. MA: logistical support, data acquisition, result discussion, and revising manuscript. All authors contributed to the article and approved the submitted version.

## Conflict of Interest

The authors declare that the research was conducted in the absence of any commercial or financial relationships that could be construed as a potential conflict of interest.

## Publisher's Note

All claims expressed in this article are solely those of the authors and do not necessarily represent those of their affiliated organizations, or those of the publisher, the editors and the reviewers. Any product that may be evaluated in this article, or claim that may be made by its manufacturer, is not guaranteed or endorsed by the publisher.
